# The Long Noncoding RNA HOTAIR in Breast Cancer: Does Autophagy Play a Role?

**DOI:** 10.3390/ijms18112317

**Published:** 2017-11-03

**Authors:** Elżbieta Pawłowska, Joanna Szczepanska, Janusz Blasiak

**Affiliations:** 1 Department of Orthodontics, Medical University of Lodz, 92-216 Lodz, Poland; elzbieta.pawlowska@umed.lodz.pl; 2 Department of Pediatric Dentistry, Medical University of Lodz, 92-216 Lodz, Poland; joanna.szczepanska@umed.lodz.pl; 3 Department of Molecular Genetics, Faculty of Biology and Environmental Protection, University of Lodz, 90-236 Lodz, Poland

**Keywords:** HOTAIR, breast cancer, autophagy, chromatin remodeling, cancer progression, miR-34a, matrix metalloproteinases, lncRNA–miRNA axis

## Abstract

HOTAIR (HOX transcript antisense RNA) plays a critical role in chromatin dynamics through the interaction with histone modifiers resulting in transcriptional gene silencing. The promoter of the *HOTAIR* gene contains multiple estrogen response elements (EREs) and is transcriptionally activated by estradiol in estrogen receptor-positive breast cancer cells. HOTAIR competes with BRCA1, a critical protein in breast cancer and is a critical regulator of genes involved in epithelial-to-mesenchymal transition. It mediates an oncogenic action of c-Myc, essential for breast carcinogenesis. The carcinogenic action of HOTAIR was confirmed in breast cancer stem-like cells, in which it was essential for self-renewal and proliferation. Several miRNAs regulate the expression of HOTAIR and HOTAIR interacts with many miRNAs to support cancer transformation. Many studies point at miR-34a as a major component of HOTAIR–miRNAs–cancer cross-talk. The most important role of HOTAIR can be attributed to cancer progression as its overexpression stimulates invasion and metastasis. HOTAIR can regulate autophagy, important for breast cancer cells survival, through the interaction with miRNAs specific for autophagy genes and directly with these genes. The role of HOTAIR-mediated autophagy in breast cancer progression can be underlined by its interaction with matrix metalloproteinases, essential for cancer invasion, and β-catenin can be important for this interaction. Therefore, there are several mechanisms of the interplay between HOTAIR and autophagy important for breast cancer, but further studies are needed to determine more details of this interplay.

## 1. Introduction

The complexity of an eukaryotic organism, which can be measured by the number of different cell types, cannot be directly correlated with the structure of its genome [1,2]. Several features of the genome are positively correlated with the complexity of an organism and the ratio of protein-coding to non-coding DNA seems to decrease with the complexity and in humans it is estimated to about 1% [3]. However, a great majority of this non-coding DNA is transcribed, producing non-coding RNA (ncRNA) [4]. Long, over 200 nt, ncRNAs (lncRNAs) are ubiquitously transcribed in the human genome, but our knowledge regarding their amount and significance in physiology and pathology is far from complete, despite many studies on these species [5]. However, some data suggest that they can be an important player in the human genome expression, especially as some of these by definition non-coding objects are translated to produce polypeptides of largely unknown functions.

Human lncRNAs play an important role in a variety of physiological processes, including epigenetic regulation of gene expression, RNA decay, microRNA regulation, RNA splicing, protein folding [6,7,8]. LncRNAs can be important in demarcating regions of active and silent chromatin, which is a major issue in epigenetics to be resolved [9]. Impaired functioning of lncRNAs can lead to disturbances of these processes resulting in serious human diseases, including cancer [10,11,12,13].

Breast cancer is globally the most serious malignancy affecting women worldwide, and there have been many experimental studies and clinical trials to improve its diagnosis and therapy. However, molecular mechanisms of its pathogenesis are still not completely known. Autophagy, a process of degrading and re-using of unneeded or damaged cellular components, recently appreciated by the Nobel Prize, is important in cancer transformation and functions of a cancer cell, including metastasis [14,15,16,17]. Many proteins important for autophagy are regulated by lncRNAs [18,19,20,21]. The importance of lncRNAs for cancer transformation concerns also breast cancer [22,23,24,25]. Moreover, breast cancer belongs to the most intensively studied malignances in respect to their regulation by lncRNAs, and HOX (homeobox) transcript antisense RNA (HOTAIR) is an lncRNA, which may play an important role in this regulation, mainly due to a wide spectrum of miRNAs interacting with HOTAIR and its effect on matrix metalloproteinases (MMPs) [26,27,28,29]. Both HOTAIR-interacting miRNAs and MMPs might be involved in autophagy regulation [15,30,31,32,33,34]. Therefore, it is tempting to consider the involvement of autophagy in the role of HOTAIR in breast carcinogenesis.

## 2. Homeobox Transcript Antisense RNA (HOTAIR)

**Figure 1:**
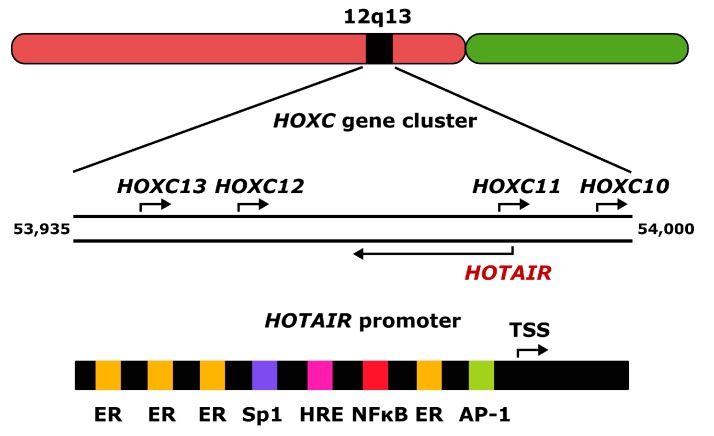
The 2158 nucleotide long human HOX transcript antisense RNA (HOTAIR) gene is located within the 12q13 chromosomal region in the HOXC (homeobox C) gene cluster and is transcribed from the antisense strand of the cluster. The promoter of the *HOTAIR* gene contains binding sites for many transcription factors, including several estrogen response elements (ER) and sites for specificity protein 1 (SP1), nuclear factor kappa B (NF-κB), activator protein 1 (AP1), hypoxia response elements (HRE). TSS–transcription start site; the upper strand of the HOXC cluster is presented in the 5′→3′ orientation as is the *HOTAIR* promoter; the numbers indicate chromosomal position of the HOXC cluster in bp; arrows show the direction of transcription.

HOX (homeobox) transcript antisense RNA (HOTAIR, primarily HOX antisense intergenic RNA) is affiliated as a 2158 nucleotide long lncRNA, whose gene is located within the HOXC (homeobox C) gene cluster at the 12q13 chromosomal region [9]. It is transcribed by RNA polymerase II from the antisense strand of the *HOXC* (homeobox C) gene cluster, capped, and polyadenylated. The human HOTAIR gene has 6 exons separated by 5 introns (Figure 1). Alternative splicing of its primary transcript results in several variants. A single promoter and 18 enhancers have been identified in the *HOTAIR* gene (http://www.genecards.org/cgi-bin/carddisp.pl?gene=HOTAIR; 1 October 2017) to date. The *HOTAIR* promoter contains binding sites for many transcription factors, including several estrogen response elements (EREs) targeted by the activated estrogen–estrogen receptor (ER) complex. Other transcription factors binding the *HOTAIR* promoter are activator protein 1 (AP1), specificity protein 1 (Sp1), nuclear factor kappa B (NF-κB), hypoxia response elements (HREs), proteins of the SRC (serine, arginine and cysteine-rich) family, and others. Therefore, HOTAIR can be involved in estrogen signaling and in consequence in estrogen-dependent cancer transformation, although the causative relationship between HOTAIR and such cancers cannot be established on the basis of the presence of EREs, as these motifs are common in the human genes.

HOTAIR can adopt a complex secondary structure, consisting of many stem and loop structures, whose occurrence depends primarily on base-pairing conditions. An excellent review on this subject was presented by Wang et al. [35].

**Figure 2:**
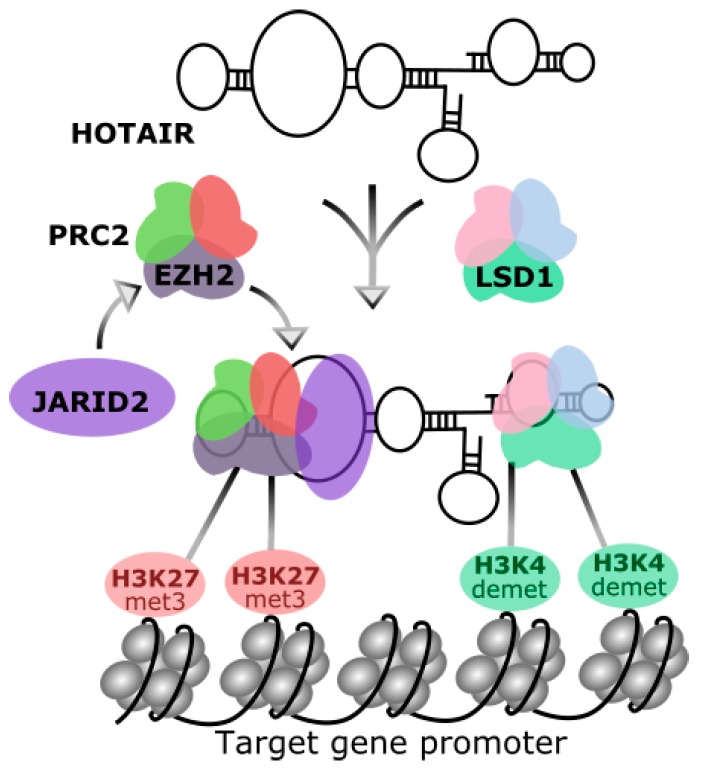
Chemical modifications of chromatin directed by HOTAIR. HOTAIR recruits polycomb repressive complex 2 (PRC2), containing the histone methyltransferase EZH2 (enhancer of zeste 2 polycomb repressive complex 2 subunit), which is activated by JARID2 (Jumonji and AT-rich interaction domain containing 2). Active EZH2 (enhancer of zeste 2 polycomb repressive complex 2 subunit) trimethylates H3 histone at lysine 27 (H3K27met3) bound to the target gene. On the other hand, HOTAIR interact with LSD1 (lysine specific demethylase 1A), a histone demethylase, which is a part of larger protein complex and demethylases H3 histone at lysine 4 (H3K4demet). These chemical modifications of histone tails lead to chromatin complexing and silencing of the transcription of target gene(s).

The primary identified function of HOTAIR was the trans-regulation of gene expression in the *HOXD* (homeobox D) locus [9]. Chemical modifications of histone N-terminal tails are a major determinant of the chromatin structure. It was postulated that activities, which are responsible for such modifications are guided by non-coding RNAs [36]. Rinn et al. provided convicting evidence that HOTAIR could be a mediator of transcriptional silencing. These authors showed that HOTAIR-bound polycomb repressive complex 2 (PRC2), which displays histone methylase activity and trimethylates H3 histone on lysine 27 (H3K27met3) targeting a region in the chromatin to be silenced (Figure 2) [9]. That methylase activity is provided by histone methyltransferase EZH2 (enhancer of zeste 2 polycomb repressive complex 2 subunit), a member of the Polycomb group family and requires stimulation by the JARID2 (Jumonji and AT-rich interaction domain containing 2) protein [37]. HOTAIR can actively recruit PRC2 or may play a role of a platform to bind for PRC2. Next, HOTAIR was shown to interact with histone demethylase LSD1 (lysine specific demethylase 1A), another chromatin modifier critical for gene silencing [38]. HOTAIR directs LSD1-mediated demethylation of histone H3 at lysine 4 (H3K4demet). LSD1 is a subunit of the LSD1–CoREST (corepressor of RE1 (repressor element-1) silencing transcription factor)–REST protein complex and is supposed to demethylate only mono- and dimethylated H3K, so the trimethylation induced by EZH2 may not be affected by its activity [38]. Therefore, HOTAIR is a modulator of chromatin scaffold and directly interacts with chromatin modifiers, PRC2 and LSD1, guide them to the target genes loci to suppress their transcription. It was shown that HOTAIR bound a DNA polypurine motif (GA-rich polypurine motif, the HOTAIR motif) regulating gene transcription, and this effect can be extended for other sites in the genome and other regulatory lncRNAs [39]. 

Recently, Portoso et al. showed that PRC2 is dispensable for HOTAIR to repress transcription [40]. These authors suggested that PRC2 binding could result from gene silencing mediated by HOTAIR. This somehow surprising hypothesis requires verification in further studies.

**Figure 3:**
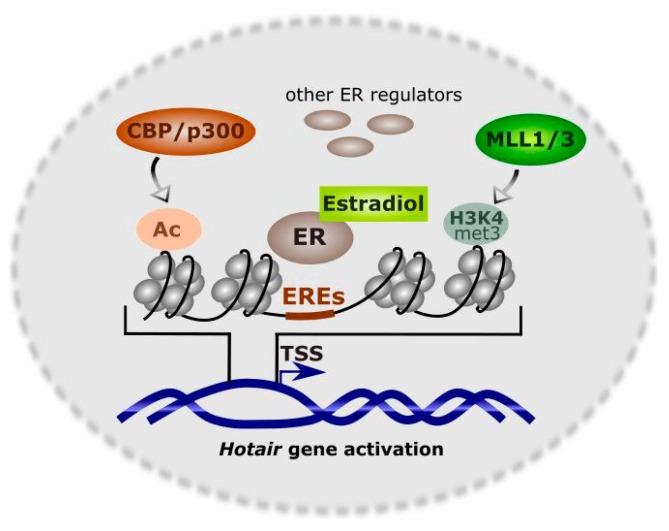
Transcriptional regulation of HOTAIR in the presence of estradiol. Several estrogen receptors (ERs) and their coregulators can bind the *HOTAIR* promoter, including the mixed lineage leukemia (MLL) family of histone methyl-transferases MLL1 and MLL3, histone acetyl-transferases CBP (cAMP response element-binding protein)/p300, and other proteins. Their action results in trimethylation of histone H3 at lysine 4 (H3K4met3) and histone tails acetylation (Ac) associated with trans-activation of HOTAIR. EREs–estrogen response elements, TSS–transcription start site.

Basal *HOTAIR* transcription is maintained by the nuclear receptor corepressor (N-CoR) complex, which is bound to the *HOTAIR* promoter in the absence of trans-activators, but their recruitment decreases the affinity of N-CoR [41,42]. The presence of several EREs implies that estrogens can play an important role in the expression of HOTAIR. Bhan et al. showed that HOTAIR expression in ER-positive breast cancer cells was transcriptionally activated by estradiol and ERs were critical for this activation [42]. Not only ERs but also their regulators are important as they can bind promoters of the gene regulated by estradiol [43]. ERs regulators include chromatin-remodeling enzymes [44]. Estradiol-dependent activation of HOTAIR can occur in various forms and mixed lineage leukemia (MLL) family of histone methyl-transferases (MLLs) are probably the most universal ER-coregulators and MLL1 and MLL3 bind the promoter of the *HOTAIR* gene in the presence of estradiol [34]. Other proteins important for estradiol-mediated transcriptional regulation of HOTAIR expression are histone acetyl-transferases CBP (REB (cAMP response element-binding protein)-binding protein)/p300. The collective action of these factor results in increased levels of H3K4met3 and histone acetylation and activation of *HOTAIR* transcription [42] (Figure 3).

HOTAIR is an lncRNA, which modulates gene expression by directing the chromatin-modifying complexes to specific sites in the genome. The best known representative of this class of lncRNAs is XIST (X-inactive specific transcript) RNA, which is involved in keeping the balance of the products of X chromosome in male and female cells [45]. The other major function of lncRNAs follows from its involvement in pattern formation and differentiation [3,46].

Apart from transcriptional regulation of gene expression, HOTAIR is involved in post-translational modifications. It was reported that HOTAIR interacted with E3 ubiquitin ligases: Dzip3 and Mex3b [DAZ (deleted in azoospermia) interacting zinc finger protein 3 and Mex-3 (muscle excess 3) RNA binding family member B, respectively] and their ubiquitination substrates, Ataxin-1 (spinocerebellar ataxia type 1 protein) and Snurportin-1 (RNA U transporter 1), respectively [47,48]. Therefore, HOTAIR can serve as a protein ubiquitination and subsequent degradation platform. It was also shown in these studies that HOTAIR was overexpressed in senescent cells and its silencing reversed cellular senescence [47]. Altogether, these results suggest that HOTAIR can modulate the cellular senescence program by proteosomal degradation of senescence-related proteins.

Many lncRNAs possess bioinformatically identified miRNA recognition elements (MREs), suggesting that the transcription of some miRNAs is regulated by lncRNAs and some lncRNAs are involved in synthesis and maturation of miRNAs, while others—in their degradation [49]. Consequently, in normal conditions there should be a functional balance between lncRNAs and miRNAs, which is known as the lncRNA–miRNA paradigm [50]. The crosstalk between lncRNAs and miRNAs can decide on the pathological state of the cell, resulting in serious human diseases. These include many cancers (bladder, breast, gastric and prostate cancers, glioma, hepatocellular, and renal carcinomas), cardiovascular diseases (atherosclerosis, myocardial infarction, maladaptive cardiac hypertrophy, and ventricular septal defect), neurodegenerative diseases (spinocerebellar ataxia, Parkinson’s, Alzheimer’s, and Huntington’s diseases), and other diseases (pulmonary fibrosis, inflammation, etc.). An excellent review on this subject has been recently published by Bayoumi et al. [51]. HOTAIR was shown to be enriched and stabilized in HeLa cervical cancer cells after inhibition of the endogenous miRNA let-71 [47]. Argonaute proteins are a key player in miRNA-mediated gene silencing [52]. HOTAIR was suppressed by a direct cleavage by Argonaute 2, RISC (RNA interference silencing complex) catalytic component 2 (AGO2), in the presence of miR-141 [53]. This effect is important for the role of HOTAIR in cancer transformation, as miR-141 in contrast to HOTAIR, is considered as a tumor suppressor, so its interaction with HOTAIR can significantly influence the process of malignant transformation, including breast carcinogenesis [54,55,56,57,58,59,60,61,62,63]. Several mechanisms can underline the interaction between HOTAIR and miR-141 and recently it was shown that miR-141 interacted with SKA2 (spindle and kinetochore associated complex subunit 2), which is essential for proper chromosome segregation and this interaction was mediated by HOTAIR [64]. It was suggested that HOTAIR and miR-196a acted synergistically and enhanced the aggressiveness of gastrointestinal tumors [65]. HOTAIR was reported to overexpress in glioma cells, and its knockdown resulted in miR-326-mediated suppression of oncogenic effects [66].

## 3. HOTAIR in Breast Cancer

Breast cancer has a special position among all malignancies for at least two reasons. First, it is the most common cancer in women worldwide, so many epidemiological, clinical, and research projects to fight this disease have been undertaken. Second, about 10% of breast cancer cases are considered to be hereditary, which, besides hereditary non-polyposis colon cancer, is the highest fraction of heredity among all cancers [67]. About half of hereditary cases have mutations in the high penetrance genes *BRCA1/2*. However, mutations in these genes are also observed in sporadic cases [68]. Therefore, mutated *BRCA1* or *BRCA2* is a main genetic determinant of breast cancer occurrence.

It was shown that BRCA1 interacted with EZH2, binding its region located between 341 and 559 amino acid (aa) and overlapping with the non-coding RNA binding domain 1 (ncRBD1) in EZH2 [69]. Because ncRBD1 is needed for the interaction of HOTAIR with EHZ2, a competition between HOTAIR and BRCA1 for overlapping regions in EZH2 was postulated. Therefore, the competition of HOTAIR with BRCA1 can influence regulation of gene expression by HOTAIR and the results of this competition can be different for different forms of mutations in the *BRCA1* gene. Consequently, HOTAIR can be an important regulator of gene expression in breast cancer associated with BRCA1 mutations.

The *HOTAIR* promoter contains several EREs, and it was shown that estradiol regulated *HOTAIR* expression in ER (estrogen receptor)-positive breast cancer cells [42]. Two of four identified EREs (Figure 1), located at −1486 and −1721 bp within the *HOTAIR* promoter were involved in that activation. However, this regulation was abolished in breast cancer cells with inactive ERs, indicating a critical role of the receptors in estradiol-mediated control of *HOTAIR* expression [42]. In line with these studies are experiments showing that bisphenol-A and diethylstilbestrol, estradiol agonists belonging to estrogenic endocrine disruptors, stimulate the expression of *HOTAIR* both in vitro and in vivo [41,42].

The promoter binding by the histone methylases MLL1 and MLL3 and CBP/p300, which are histone remodeling factors in an E2-dependent manner can be important for the epigenetic functions of HOTAIR in breast cancer [42]. It was postulated that HOTAIR in its basic state is maintained by a coordinated action of N-CoR and mixed lineage leukemia 2 (MLL2), but E2 can activate *HOTAIR* promoter by protein–protein interaction resulting in chromatin remodeling and gene expression [42].

A meta-analysis suggests that HOTAIR is important for lymph-node metastasis, which is an early, clinically detected step of tumor cell dissemination in most cancers, of special importance in breast cancer, as in this disease, manual control of axillary lymph nodes is a routine [70].

Analysis of HOTAIR and other lncRNAs expression in 164 cases of ER-positive primary breast cancer led to the conclusion that they could be an independent prognostic marker in ER^+^ breast cancer, as their higher expression was correlated with a worse prognosis [71,72]. Therefore, HOTAIR can be important for breast cancer progression, as it was shown that HOTAIR expression increased in primary breast cancer and metastases and the level of HOTAIR expression in primary tumors was a powerful indicator of metastasis [73]. That work suggests a more general conclusion that lncRNAs in the HOX loci are constantly dysregulated during breast cancer progression. The metastasis-promoting action of HOTAIR was strictly associated with changes in PRC2 functioning, leading to genome-wide epigenetic alterations. Importantly, loss of HOTAIR resulted in a decreased invasiveness of breast cancer cells, especially in cells with overactive PRC2. These important studies suggest a significant role of HOTAIR in breast cancer pathogenesis and its large potential in diagnosis and therapy of this disease.

Lu et al. studied HOTAIR expression and methylation of its downstream intergenic CpG islands in 348 samples of primary breast cancer [74]. Although both quantities varied greatly in the studied population, a positive correlation between HOTAIR expression and CpG methylation was observed. Increased methylation was associated with a worse prognosis for the patient. The level of HOTAIR expression was not associated with clinical characteristics of breast cancer in a multivariate analysis, but an association between enhanced HOTAIR expression and lower disease relapse and mortality was found in a univariate analysis. These results suggest that HOTAIR may not be an independent breast cancer marker and requires further research.

The promoter of the HOTAIR gene can be bound by interferon regulatory factor-1 (IRF1), which inhibits HOTAIR activity in cancer [75]. In turn, decreased Akt activation by its decreased phosphorylation increased IRF1 and diminished HOTAIR expression in MCF-7 breast cancer cells [76]. 

The c-Myc oncoprotein regulates transcription in breast cancer cells through another oncoprotein, HBXIP (hepatitis B X-interacting protein), which binds the promoter of its gene [77]. c-Myc recruited HOTAIR and LSD1. Therefore, the HBXIP/HOTAIR/LSD1 complex can be critical for transactivation by c-Myc in breast cancer and possibly for general oncogenic function of this oncogene.

Bhan et al. showed that HOTAIR is critical for survival and proliferation of MCF-7 breast cancer cells [42]. Knockdown of HOTAIR resulted in a suppression of MCF-7 growth and led to their apoptosis. As the knockdown induced upregulation of many other genes important for cancer transformation, it was concluded that the role of increased expression of HOTAIR in tumorigenesis could result from its inhibition of tumor suppressors.

A correlation between the expression of EZH2 and HOTAIR in breast cancer was observed in a high throughput tissue microarray study [78]. However, high HOTAIR expression was associated with positive ER and PR (progesterone receptor), while high expression of EZH2 was linked with an increased proliferation rate, ER and PR negativity, lower HER2 (human epidermal growth factor receptor 2) expression and triple negativity. Both HOTAIR and EZH2 showed a higher expression level in primary breast cancer samples than metastases.

Padua Alves et al. showed that HOTAIR was a critical regulator of genes involved in epithelial-to-mesenchymal transition (EMT) in a breast cancer cell line, and this role was supported by transforming growth factor β1 (TGF-β1) [63]. EMT is a critical step in cancer invasion and metastasis.

This action was underlined by a direct inhibition of the SETDB1 (SET Domain Bifurcated 1) oncogene by miR-7, which was associated with a downregulation of the STAT3 (signal transducer and activator of transcription 3) pathway. Therefore, HOTAIR can be important for the aggressiveness of breast cancer, so it can be considered as a target in the therapy of highly invasive cases of this disease. These studies are in agreement with results showing a critical role of HOTAIR in maintaining the nuclear factor of activated T cells 5 (NFAT5) in breast cancer [79]. NFAT5 is a transcription factor playing an important role in cancer cell migration and metastasis. This important function of HOTAIR resulted from the suppression of miR-568 underlined by H3K27 methylation and H3K4 demethylation on the miR-568 loci. HOTAIR-mediated upregulation of NFAT5 resulted in overexpression of the calcium-binding protein S100A4 and vascular endothelial growth factor C (VEGF-C). These studies suggest another mechanism of HOTAIR in breast cancer metastasis and therefore another strategy of HOTAIR-related antimetastatic therapy in this disease.

HER2 is an important player in breast carcinogenesis and its overexpression, associated with a bad prognosis, is observed in about one third of all breast cancer cases [80]. It is targeted by many miRNAs and HOTAIR can recruit some of them, including miR-34a, as shown in gastric cancer [81]. That study justified a further study on HOTAIR–ER2 interaction in breast cancer. HOTAIR was shown to co-express with *FOXA1* (forkhead box A1) and *FOXM1* (forkhead box M1) in HER2-enriched breast tumors. These studies provided details on transcriptional regulation of HOTAIR and its prognostic value in breast cancer, especially in endocrine therapy in ER2-positive breast cancer cases [82]. 

When targeting the HOTAIR–miRNA axis in breast cancer therapy, it is important to limit the number of miRNAs to those, which are specific or enriched in this disease. Therefore, miRNAs overexpressed/enriched in the breast are primary candidates as their targeting could not affect the function of the HOTAIR–miRNA axis in non-cancer tissues. This is a general feature of anticancer strategy targeting miRNAs [83].

Circulating DNA of the *HOTAIR* gene is considered as a marker in breast cancer, which was shown in a population-based study [84]. In addition, a positive correlation between the level of this DNA and clinical stage of breast cancer was observed in that research.

HOTAIR was shown to enhance therapeutic resistance to ionizing radiation in breast cancer MDA-MB-231 cells associated with increased proliferation and activation of the Akt pathway [85].

As cancer stem cells (CSCs) are responsible for maintaining tumor cells population and play a major role in cancer metastasis, reinitiating, therapeutic resistance and transplantation, it is justified to look for a possible role of HOTAIR in breast CSCs [86]. HOTAIR was shown to inhibit miR-7 in breast CSCs, which in turn inhibited cell invasion and metastasis of breast CSCs xenograft, EMT, and breast CSCs density [87]. Deng et al. obtained a cancer stem-like cell population from breast cancer cell line MCF7 or MDA-MB-231 on the basis of self-renewal capacity and tumor formation in vitro and in vivo [88]. In this study, HOTAIR was the only lncRNA, which was overexpressed in both CSC subpopulations as compared with their non-stem counterparts among several studied lncRNAs. HOTAIR regulated proliferation, colony formation, migration, and self-renewal of CSCs obtained from MCF-7 line and an interaction with miR-34a contributed to these effects. Moreover, transcriptional control of miR-34a by HOTAIR influenced SOX2 [SRY (sex determining region Y)-Box 2], an essential stemness factor regulating self-renewal capacity of CSCs. Upregulation of HOTAIR affected proliferation and colony formation in CSCs by induction p53 expression in the p53/p21 pathway, which is a basic cell cycle control component. This important work not only confirms earlier reports on the importance of HOTAIR in breast cancer transformation and its clinical usefulness in diagnosis, prognosis, and therapy, but also shows many details of the mechanism of HOTAIR action. Moreover, it also confirms a significant role of stem-like cells in cancer transformation. These studies point at miR-34a as an important element of HOTAIR regulation, which was suggested earlier [89]. Similarly, HOTAIR was reported to indirectly regulate miR-34a expression [82].

## 4. Autophagy and Its Role in Cancer

Autophagy is the main intracellular system to degrade unnecessary and damaged material (cargo) in the lysosome. The process of delivery of the cargo to the lysosome is called autophagic flux. Degraded material is further recycled to provide the cell new building blocks and energy. Therefore, autophagy is a mechanism of renovation of cells and tissues [90].

**Figure 4:**
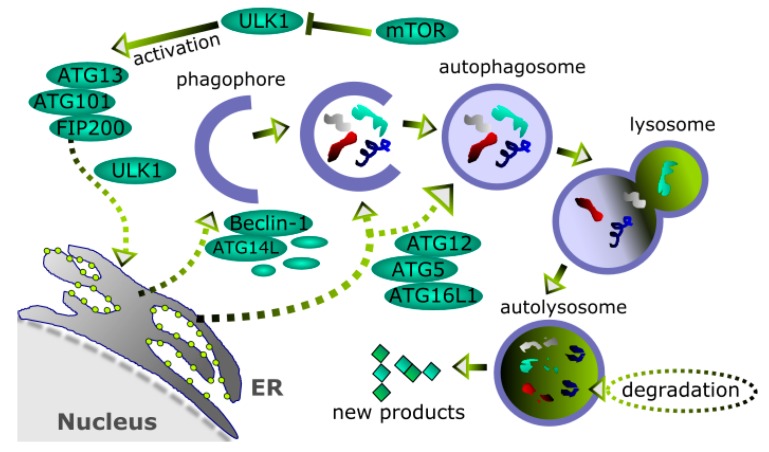
Autophagy. Unnecessary or damaged cellular material, including organelles, trapped in a nucleating endoplasmic reticulum (ER)-related membrane (phagophore) to form a closed vehicle (autophagosome) is delivered to lysosome, which fusions with autophagosome, giving autolysosme, in which the material is finally degraded and recycled. Basically, autophagy can proceed as an mTOR (mammalian target of rapamycin)-dependent, as presented in this figure, or -independent mechanism. Many proteins are involved in autophagy, including autophagy-related (ATG) proteins, Beclin-1, which suppresses ULK1 (Unc-51-like autophagy activating kinase 1) complex, and LC3 (microtubule-associated protein 1A/1B-light chain 3). Sharp arrows—activation, blunt arrows—inhibition, dashed short arrows—possible, not always occurring routes.

Usually, autophagy is classified into macroautophagy, microautophagy, and chaperone-mediated autophagy. Macroautophagy, commonly referred to as autophagy, is characterized by an intermediate organelle in the form of a membrane vehicle called autophagosome preceded by the formation of an isolation membrane (phagophore) (Figure 4). Autophagy can be induced by many factors, including a variety of stress conditions. Autophagosomes are formed on or close to endoplasmic reticulum (ER) [91]. Autophagosome formation is directed by many autophagy-related (ATG) proteins [92]. The mTOR (mammalian target of rapamycin)-dependent autophagy is a major autophagic mechanism in humans, in which the mTOR protein suppresses ULK1 (Unc-51 like autophagy activating kinase 1) complex, an autophagy activator [93]. Apart from the ULK1 protein, the complex contains ATG13 and 101 as well as FIP200 and upon activation is transported to ER, where it regulates another complex including Beclin-1, ATG14L, and other proteins, involved in the formation of isolation membrane. The extension and closure of the isolation membrane is supported by the conjugate of LC3 (microtubule-associated protein 1A/1B-light chain 3) with phosphatidylethanolamine (PE) associated with the ATG12–ATG5–ATG16L1 complex.

Autophagy is important for many physiological processes and its impairment is associated with serious human pathologies, including cancer. However, it is not easy to determine when autophagy has a beneficial impact on the cell and when such an impact is detrimental. This is due to two opposite pathways of autophagy—pro-survival and pro-death [94]. They can be useful in cancer therapy, when cancer cells are targeted, but normal cells should be protected. However, this is a rather complex issue, which is a challenge in general cancer therapy, but autophagy singly can also be targeted as some cancer cells can be killed by inhibiting autophagy and other cells—by its stimulation [95].

Emerging evidence suggests an involvement of the lncRNA–miRNA axis in autophagy [20,96,97,98]. It seems that it can be important in regulating mTOR signaling and modulating ATGs. FLJI1812, an lncRNA originating from 3′-UTR (untranslated region) of the transforming growth factor β2 (*TGFβ2*) gene, is processed by T-cell-restricted intracellular antigen-1 (TIA1), which in turn requires phosphorylation mediated by mTOR activation [99]. FLJI1812 competed with miR-4459 on its target, *ATG13*, resulting in an increased autophagy. The results of similar studies showed that downregulation of HOTAIR in endometrial cancer cells resistant to the anticancer drug cisplatin resulted in decreased proliferation and increased autophagic activity in these cells [100]. HOTAIR was reported to promote proliferation of hepatocellular carcinoma through activation of autophagy by overexpression of *ATG3* and *ATG7* [101].

Knockdown of HOTAIR inhibited autophagy in a chondrosarcoma cell line [102]. The mechanism of this effect involved HOTAIR-induced miR-454-3p DNA methylation by recruiting EZH2 and DNA Methyltransferase 1 (DNMT1) to its promoter. This resulted in silencing of miR-454-3p and subsequent reduction of autophagy, as the *ATG12* gene, encoding a protein essential for autophagy, was among targets of miR-454-3p.

An important role of miR-34a in carcinogenic action of HOTAIR can be related to autophagy. Liu et al. observed that miR-34a inhibited autophagy in acute myeloid leukemia by targeting the HMGB1 (high-mobility group box 1 protein), which interacts with Beclin-1, playing a central role in autophagy and is considered as critical for this process [103,104]. A similar effect has been observed in retinoblastoma cells [105]. Another mechanism of miR-34a-mediated suppression of autophagy was highlighted by Song et al., who showed that this effect resulted from the interaction of miR-34a with FoxO3 (Forkhead Box O3) in mouse alveolar type II epithelial cells in acute lung injury [106]. FoxO3 is an important regulator of cell death and its impaired expression is associated with lung cancer [107]. Liu et al. showed that miR-34a inhibited autophagy in mouse tubular epithelial cells in acute kidney injury, binding 3′-UTR in the *ATG4B* gene [108]. The gene encoding ATG9 was reported to be regulated by miR-34a in *Caenorhabditis elegans* [109]. Therefore, there is accumulating evidence on the role of miR-34a in autophagy regulation, and this role was confirmed in breast cancer. Li et al. observed that the endogenous protein kallistatin induced autophagy in the breast cancer cell line MDA-MB-231, demonstrated by an enhanced level of LC3B as well as increased expression of ATG5 and Beclin-1 [110].

## 5. Do HOTAIR and Autophagy Interplay in Breast Cancer?

A deletion of one allele of the *Beclin-1* gene was observed in breast cancer and other estrogen-dependent malignancies: ovarian and prostate cancers [111].

The expression of the lncRNA *ROR* (regulation of reprogramming) gene was higher in samples taken from tumors obtained from breast cancer patients than in adjacent regions [18]. These studies on clinical material were supplemented with research on the human breast cancer cell line BT474. It was observed that the downregulation of *ROR* abolished drug resistance to tamoxifen through induction of autophagy, manifested by an increased expression of *LC-3* and *Beclin-1*.

B-type Eph receptor 2 (EphB2) is a developmentally regulated Eph-related tyrosine kinase, which plays a role in normal development and cancer [112]. Although it was expressed in benign breast tumor, its expression was much higher in breast cancer samples [113]. This increased expression was associated with induction of autophagy as assessed by immunostaining of LC3, ATG5, and ATG12. Furthermore, EphB2 upregulated metalloproteinases 2 (MMP2) and MMP9 in a breast cancer line.

Cancer progression requires degradation of the extracellular matrix (ECM), which is mediated by various enzymes, including matrix metalloproteinases [114,115]. Therefore, the interaction of HOTAIR with MMPs may partly underline its role in cancer progression. This assumption was confirmed by Qiu et al., who showed an increased expression of *HOTAIR* in ovarian cancer, resulting in the upregulation of MMP-9 and MMP-3, contributing to increased cellular proliferation, migration, and invasion of ovarian cancer cells [116,117]. These studies are in line with research showing a downregulation of MMP9 and a reduction in proliferation in the hepatocellular cell line bearing HOTAIR knockdown [118]. A decrease in migration and invasiveness of breast cancer MCF-7 cells was associated with a decreased expression of MMPs 2 and 9 in these cells, in which HOTAIR was downregulated by siRNA [28]. In addition, knockdown of *HOTAIR* resulted in G1 arrest and decrease in the expression of the p53/Akt/JNK pathway in MCF-7 cells. Upregulated HOTAIR induced overexpression of MMPs in other diseases as well, as shown in temporomandibular joint osteoarthritis, in which HOTAIR-induced overexpression of MMP 1, 9, and 3 was mediated by IL-1β (interleukin 1β) [119].

The MMP–HOTAIR axis can be associated with the role of HOTAIR in cancer progression. Detrimental action of MMPs may be suppressed by autophagy as shown in endothelial cells treated with a high concentration of glucose [120]. However, an increase in certain MMPs cannot be unequivocally associated with ECM degradation in cancer, as this process is mediated by many enzymes, and even MMPs action depends on the balance between MMPs and their inhibitors [121,122]. It was observed that timosaponin AII, a steroidal saponin tested as an anticancer drug, induced autophagy in cancer cell lines [123]. This effect was associated with an inhibition of MMP-2 and -9 via suppression of the ERK1/2, Src/FAK and β-catenin signaling pathways. As mentioned earlier, HOTAIR promoted activation of the Wnt/β-catenin pathway in esophageal carcinoma, so β-catenin can link HOTAIR, MMPs, and autophagy in cancer.

## 6. Conclusions and Perspectives

HOTAIR plays an important role in epigenetic regulation of gene expression through the modification of chromatin structure leading to gene silencing. It is also important for functional demarcation of the region of transcriptionally active and inactive chromatin, being a scaffold for histone modifiers. Its overexpression is associated with many cancers and in fact, *HOTAIR* is a proto-oncogene and is associated with cancer progression and bad prognosis. The promoter of its gene contains sites for estrogen receptor binding, suggesting that the role of HOTAIR in malignant transformation can be especially important in estrogen-dependent cancers, including breast cancer. Identification of the competition between HOTAIR and BRCA1, a critical protein in breast cancer, supports this assumption.

Although many reports suggest the clinical utility of HOTAIR as an independent marker in breast cancer, further studies on this subject are needed, as it can be directly related to patients’ health. Moreover, some reports do not support such utility of HOTAIR. Gökmen-Polar et al. stated that the utility of HOTAIR as a prognostic marker in breast cancer is limited to ER-negative cases [124]. Moreover, the value of HOTAIR in such patients can be different for lymphatic and hematogenous metastases.

Although breast cancer belongs to the most intensively studied malignancies, many aspects of its pathogenesis remains unclear. Several reports suggest that impaired autophagy can play a role in this disease. As autophagy is a basic pathway for removing unneeded cellular elements, including degraded and damaged proteins, and HOTAIR was shown to be involved in protein ubiquitination and degradation in the proteasome, it is tempting to speculate that HOTAIR can influence breast cancer transformation through its involvement in autophagy. Such involvement can follow from the balance between proteasomal and lysosomal degradation systems to remove and recycle damaged proteins that are no longer needed. In fact, several associations between HOTAIR and autophagy have been reported. However, autophagy itself is not a completely known and understood process and the question about the role of autophagy in cancer, including breast cancer, has not yet been fully answered. In general, normal autophagy can suppress cancer transformation, and impaired autophagy can stimulate it. On the other hand, defective autophagy, along with certain anticancer drugs, accelerate cancer cell death [125,126]. Therefore, future research on the role of autophagy in HOTAIR regulation of breast carcinogenesis can contribute to our general knowledge on autophagy and the role of this process in cancer. This will support an emerging role of lncRNAs in autophagy regulation [21].

Further studies on HOTAIR, autophagy, and breast cancer should focus on the role of the lncRNA–miRNA axis in HOTAIR regulation and breast cancer pathogenesis. It is particularly interesting to check whether HOTAIR may regulate the expression of *ATG* genes through a mechanism that includes a competition of HOTAIR with miRNAs binding these genes—the sponge effect [101]. In light of research performed so far, miR-34a seems to be the most important element of the lncRNA–miRNA axis to regulate HOTAIR, and further research on this species could provide information confirming such a role, but other elements of the complex regulatory miRNA network should be explored, as they can constitute the most important group of HOTAIR regulators. On the other hand, HOTAIR can directly interact with autophagic proteins, which has been shown for ATG3, ATG6, and other proteins. However, the great number of proteins involved in autophagy create ample opportunities for new research to establish a network of HOTAIR–autophagy interactions.

**Figure 5:**
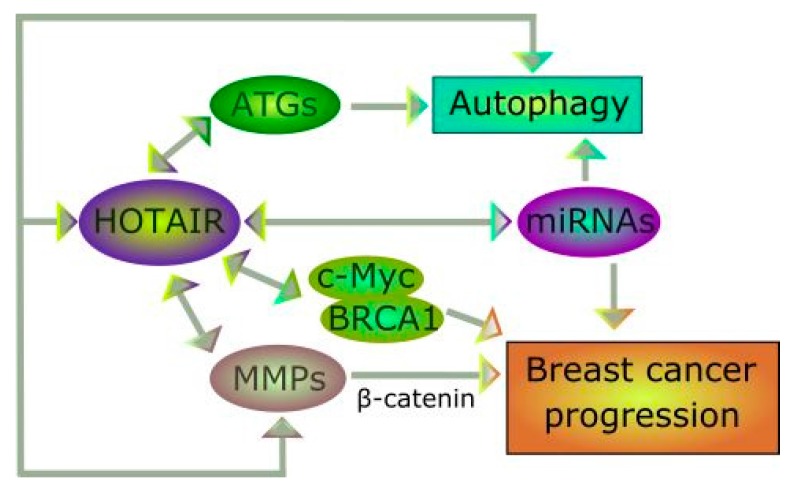
The HOTAIR–breast cancer–autophagy cross-talk. HOTAIR regulates many miRNAs and several miRNAs can be important for HOTAIR functions. These miRNAs include species important for autophagy regulation and cancer progression, but these processes can be regulated by HOTAIR in different pathways involving interaction with autophagy-related (ATG) proteins and matrix metalloproteinases (MMPs), respectively. Many other proteins can be involved in this cross-talk, including the c-Myc and BRCA1 oncoproteins, essential for breast cancer.

Matrix metalloproteinases are important for cancer progression and can play a role in the autophagic regulation of cell homeostasis. Reports on the involvement of HOTAIR regulation of MMPs in a breast cancer cell line justify further studies aimed at details of this regulation, especially the involvement of β-catenin (Figure 5).

In summary, autophagy can play a role in the involvement of HOTAIR in breast cancer pathogenesis, particularly in the regulation of cancer progression. In light of results obtained so far, some matrix metalloproteinases and miRNAs important for breast carcinogenesis could be the main mediators of HOTAIR–breast cancer–autophagy.
